# Effect of Roasting Process on Total Phenolic Compounds and γ-tocopherol Contents of Iranian Sesame Seeds *(Sesamum indicum)*

**Published:** 2013

**Authors:** Behrooz Jannat, Mohammad Reza Oveisi, Naficeh Sadeghi, Mannan Hajimahmoodi, Masoomeh Behzad, Bahman Nahavandi, Shirin Tehrani, Fatemeh Sadeghi, Morvarid Oveisi

**Affiliations:** a *Food and Drug Laboratory Research Center, Ministry of Health and Medical Education, Tehran, Iran. *; b *Department of Drug and Food Control, School of Pharmacy, Tehran University of Medical Sciences, Tehran, Iran. *; c *Islamic Azad University of Pharmaceutical Sciences, Tehran, Iran. *

**Keywords:** Roasting temperature and time, Sesame seeds, *γ*-Tocopherol, Total phenol compounds

## Abstract

Sesame (*Sesamum indicum *L.) seed and oil have long been used widely as healthy foods to supply energy and prevent aging. Some of the main active anti-oxidative constituents in sesame seeds are *γ*-tocopherol and phenols. The purpose of this study was to investigate the relationship between roasting temperature and time with *γ*-tocopherol and total phenolic compounds (TPC) of sesame seeds when roasted in a domestic electric oven.

Eight cultivars of sesame seeds in this study were *Darab*, *Dezful*, *Karaj*, *Moghan*, *Naz- Branching*, *Naz-NonBranching*, *Siah *and *Varamin*. Each cultivar was divided into ten group based on the roasting time (10, 15 and 20 min) and temperatures (180, 200 and 220 °C)andunroasted one. The high-performance liquid chromatography (HPLC) and spectrophotometeric methods were used for *γ*-tocopherol (n = 80) and TPC (n = 80) analysis, respectively.

The *γ*-tocopherol content ranged from 329 ± 5 mg/L in *Naz-Branching *sesame oil to 1114±7 mg/L in *Siah *sesame oil and 169±6 to 577±1 mg/kg in sesame seed respectively. *γ*-tocopherol content of six cultivars increased significantly (p < 0.05) as the roasting temperature and time; until 200 °C for 10 min, but they were decreased by roasting at 220 °C in longer time.

Also TPC increased significantly as the roasting temperature. The amount of TPC varied in different sesame cultivars from 20.109 ± 3.967 μM to 129.300±3.493 in *Varamin *and *Naz- Branching *sesame seed cultivars, respectively, also TPC increased from 70.953 ± 5.863 μM in unroasted *Naz-Branching *sesame seed to 129.300 ± 3.493 μM after roasting in 200 °C for 20 min.

The present study showed that Iranian sesame seed can be considered as a good source of natural antioxidant specially after roasting. The optimum temperature and time roasting to obtain the most *γ*-tocopherol and total phenolic content was 200 °C for 10 and 20 min, respectively.

## Introduction

Sesame (*Sesamum indicum *L.) seed and oil have long been used widely as healthy foods to provide nutraceuticals and nutrients, increase energy and prevent aging. Sesame is a good source of edible oil and widely used in cooking foods and confectionery products. Many studies have been conducted to investigate the health-promoting effect of sesame. A combination of a number of minor constituents such as tocopherols and phenolic components in the sesame seed oil may have a synergistic action in increasing the antioxidant activity against diseases caused by oxidative stress. Some of the main active anti-oxidative constituents in fresh sesame oil extracted from roasted seeds are *γ*-tocopherol and also phenols (1-3).

The primary and secondary products of lipid oxidation are detrimental to health. Excess production of free radicals makes lipid peroxides and reactive oxygen species, which leads to many harmful biological changes, such as DNA damage, aging, heart disease and also cancer (4-6). Therefore, antioxidants are at the point of attention for health professionals. The addition of antioxidants is one of the approved methods for increasing shelf-life of lipids and lipid-containing foods. Butylated hydroxy anisole (BHA) and butylated hydroxy toluene (BHT) are widely used as synthetic antioxidants in many countries, but they are reported to be carcinogenic (7). Thereafter, attempts to find natural antioxidants have become important in recent years. Tocopherols are sometimes added to the oil to decrease the oil oxidation during frying (8).

About 40 years ago, sesame seed oil was shown to be a high stable against oxidation compared to other plant oils (9). Sesame oil prepared from roasted sesame seeds has not only a distinctive flavor but also have a longer shelf-life (10-11). A higher roasting temperature has usually been used to obtain a strong flavor, but it results in a sesame oil of poorer quality (12). Therefore, to maintain good quality and high nutrient value of sesame, the optimum roasting condition should be established.

The high content of *γ*-tocopherol and phenolic components in Persian sesame seed (13-14) is the best reason to choose it for research about heat processing effect on nutraceuticals. So the purpose of the present research study was to investigate the relationship between roasting temperature and the time with *γ*-tocopherol and total phenolic content (TPC) of sesame seeds when roasted in a domestic electric oven.

## Experimental

*Sesame seeds*

Eight species of sesame seeds (*Sesamum Indicum L*.) used in this study were *Darab*, *Dezful*, *Karaj*, *Moghan*, *Naz-Branching*, *Naz-NonBranching*, *Siah *and *Varamin *and all were donated from Seed and Plant Improvement Institute (Tehran, Iran). The sesame seeds were sealed in a dark glass bottles and stored at 4~8°C until used. Each sesame seed cultivar was divided into ten groups based on unroasted ones and roasting time (10, 15 and 20 min) and temperature (180, 200 and 220 °C) of experimental method.

*Reagents and standards*

Chemicals such as n-Hexane, Methanol and Butanol were of analytical grade (Merck, Darmstadt, Germany) and were used without further purification. The standard *γ*-tocopherol (Fluka, Steinheim, Germany) was dissolved in n-Hexane to prepare a stock solution (1000 μg/mL) and stored at -4 °C in a dark refrigerator. The standard *α*-tocopherol (Merck, Darmstadt, Germany) was used as internal standard. Two grade distilled water obtained from the Distiller Set (Buchi, Swiss) was used to prepare a HPLC solvent. Folin-ciocalteau reagent, sodium bicarbonate solution and methanol 50% v/v were used for total phenolic content assay. 

*Apparatus*

High-performance liquid chromatography (HPLC) analysis was performed with an analytical HPLC set included a HPLC pump (Maxi-Star K-1000 from Knauer, Berlin, Germany), a degasser (Knauer, Berlin, Germany), a Mixing chamber (Knauer, Berlin, Germany), an auto sampler (Triathlon, Spark, AJ Emmen, The Netherlands), an Eurospher100 (4.6 mm × 25 cm) C8 Column (Knauer, Berlin, Germany), a UV spectrophotometer detector (Knauer, Berlin, Germany), a computer software (EuroChrom 2000 Version 1.6 from Knauer, Berlin, Germany) as integrator. Analytical scale (Sartorius, Germany) was in a weight range of up to 10-5 g and a Mixer (Erwatt, Italia) device was used to fine roasted sesame seeds. The centrifuge (Heraeus Germany) and UV-visible spectrophotometer (cintra 40 model) were used in TPC analysis. 

*Roasting*

All sesame seed groups were placed in a Pyrex Petri dish (8.0 cm diameter) covering with the other Pyrex Petri dish and then roasted at 180, 200 and 220 °C for 10, 15 and 20 min respectively in an electric oven. After roasting, the seeds were allowed to cool at room temperature, fined with the mixer. 

*γ-tocopherol analysis*

*Oil extraction*

A number of 2 g of roasted sesame seeds were oil extracted with Soxhlet extraction apparatus for six hour with the n-hexane as a solvent. After oil extraction, the bottle of oil extracts was kept under the hood until the solvent was evaporated. Each extracted oil was sealed in a dark glass bottles and also stored at 4~8 °C prior to HPLC analysis.

*Standard preparation*

Seven standard solutions of 0.25, 0.5, 1, 2.5, 5, 7.5 and 10 μg/mL *γ*-tocopherol with the 15 μg/mL *α*-tocopherol to be used as internal standards were prepared.

*HPLC method*

Two microlitres of the roasted sesame seed oils (n = 80) diluted with n-hexane in a 1 mL volumetric flask and then injected to the HPLC column with an auto sampler to analysis. This analysis was carried out by injecting 100 μL aliquots of a diluted oil on the Eurospher 100 (4.6 mm × 25 cm) C8 Column. The column was eluted with the mobile phase (Methanol, Butanol and Deionized water in percent of 90:6:4 v/v) at a flow rate of 1 mL/min. Thus, it had a 5 min *γ*-tocopherol kept time and also 6:30 min *α*-tocopherol kept time at 8.1 to 8.9 mega pascal (MPa) pressure. The *γ*-tocopherol and *α*-tocopherol were monitored with a UV spectrophotometer detector set at 294 nm. The amount of each sample was calculated by comparison to the area under the curve (AUC) of the standards.

*TPC analysis*

*Sample preparation*

Two hundred milligrams of roasted sesame seeds (n = 80) were extracted for 2 h with 2 mL of 50% methanol v/v at room temperature on an orbital shaker set at 200 rpm. The mixture was centrifuged at 1000 g for 15 min and the supernatant was decanted in to 4 mL vials. The pellets were combined and used for total phenolic content assay. 

*Colorimetric method*

Total phenolic content was determined calorimetrically using Folin-Ciocalteau reagent as described by Velioglu *et al. *(15). The extract (100 μL) was mixed with 1.5 mL of Folin-Cioalteau reagent and allowed to stand at 22 °C for 5 min. A 1.5 mL sodium bicarbonate solution (60 g/L) was added to the mixture. After 90 min at 22 °C, absorbance was measured at 725 nm using a UV-visible spectrophotometer.

*Statistical analysis*

Each obtained value of eight sesame sample groups (n = 160) was the mean of three determinations. Comparison between each separated sesame cultivar and also between all sesame cultivars experimental data were computed with one-way ANOVA test in SPSS (Release 16.0.1) software. Significant differences among treatment means were separated by using Dunnett’s T3 on post-hoc Multiple Comparison, at a level of p *< *0.05. BoxPlot graphs were inserted from SPSS software. Standard Deviations (SD) and also Coefficient of Variation (CV %) were computed with Excel (Microsoft Office Excel 2007) software.

## Results and Discussion

*HPLC method*

*Linearity and analytical range*

The correlation between the peak area ratios and the *γ*-tocopherol concentrations was evaluated over the range of 0.25-10 μg/mL and was found to be linear (y=0.5102x-0.139; R2=0.998; n=8). 

**Figure 1:**
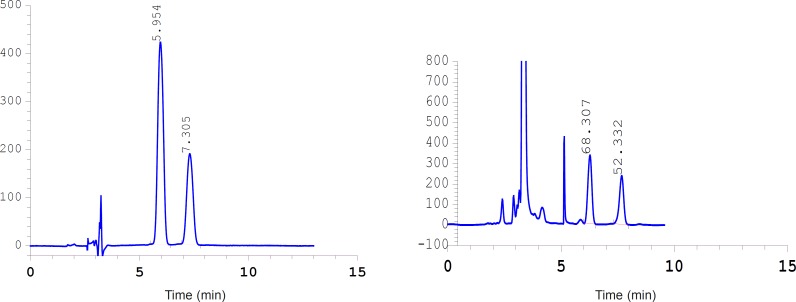
Chromatogram of *γ*-tochopherol and *α*-tochopherol acetate in: a: Calibration standard solution and b: Non branching Naz cultivar sample

*Accuracy*

The accuracy of the method was verified by means of recovery assay. This was accomplished by analyzing standard solution and spiked (enriched) sample. The analytical recovery was 99.7% for *γ*-tocopherol. 

*Precision*

The repeatability of the method was calculated by using the measured data from 3 successive HPLC injections and also on 3 successive days. Values were expressed by Coefficient of Variation (CV %). Some method validation data, including precision, sensitivity and linearity range are shown in Table 1. The data of validation method showed that the HPLC is a suitable method for *γ*-tocopherol analysis.

**Table 1:** The precision, sensitivity and linearity range of HPLC method

Precision (CV %)			
**Standard concentration**	**Repeatability**		**Internal reproducibility **
**(μg/mL)**	**(n=3)**		**(n=3)**
Standard 0.5	0.893		3.138
Standard 2.5	0.666		5.599
Standard 5	0.679		7.256
Sample *Siah *(200 °C 10 min)	0.365		1.937
Sensitivity and linearity range			
Calibration (n=6)	Range (R^2^)	Slope	Intercept
0.25-10 μg/mL	0.998	0.5102	-0.139

*Roasting effects on γ-tocopherol concentration*

The γ-tocopherol contents of eight sesame groups (n = 80) are presented in Table 2 as mg/kg. The *γ*-tocopherol content ranged from 329.5 ± 4.5 mg/L in *Naz-Branching *sesame oil to 1114±7.1 mg/L in *Siah *sesame oil and 169 ± 2.3 to 577 ± 1.3 mg/kg in sesame seed, respectively. In a number of 6 cultivars, *γ*-tocopherol content increased significantly (p < 0.05) with the rise in roasting temperature and time; until 200 °C for 10 min, but it was then decreased by roasting at 220 °C for longer time. 

**Table 2:** The *γ*-tocopherol content of eight sesame seed cultivars (n=80), roasted for different time durations (10, 15 and 20 min) and temperatures (180, 200 and 220 °C).

**Roasting Temperatur**e **(**°**C)**	**Roasting Time**	**Darab**		**Dezful**	**Karaj**	**Moghan**	**Naz-Branching**	**Naz-NonBranching**	**Siah**	**Varamin**
	**(Min)**	**(mg/kg)**	**(mg/kg)**		**(mg/kg)**	**(mg/kg)**	**(mg/kg)**	**(mg/kg)**	**(mg/kg)**	**(mg/kg)**
0		273.2	262.1		446.1	417.6	212.6	210.9	424.2	387.1
180	10	269.5	200.8		394.1	371.3	168.9	194.9	383.8	391.3
	15	215.9	225.2		419.8	457.7	189.4	183.7	544.1	467.2
	20	252.5	247.6		443.4	472.5	184.3	240.6	487.7	462.1
200	10	278.5	278.3		446.4	539.8	236.1	164.1	488.7	475.1
	15	216.2	256.8		383.8	458.2	194.7	210.4	475.9	400.4
	20	300.3	250.0		267.0	428.7	198.0	269.4	559.6	364.4
220	10	336.1	266.9		271.8	366.6	208.6	223.7	476.1	355.5
	15	344.7	269.3		325.0	270.5	181.6	205.4	450.2	384.9
	20	292.5	249.2		336.5	299.8	227.7	246.0	576.9	384.3

**Figure 2:**
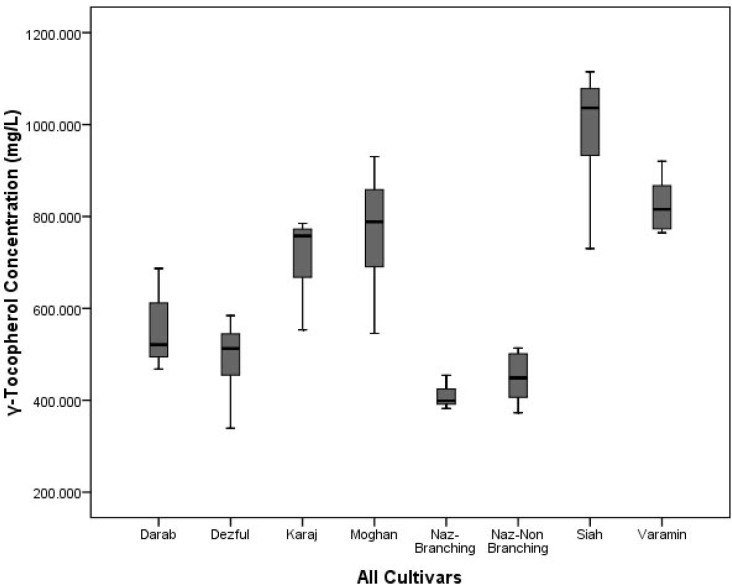
The *γ*-tocopherol content of eight sesame oil cultivars (n = 80)

A systematic comparison among the *γ*-tocopherol content of eight sesame oil cultivars (n = 80), roasted at different time and temperatures in the range of 10 to 20 min and 180 to 200 °C is shown in Figure 2. As it shows, the *Siah *cultivar has the most *γ-*tocopherol content among the whole cultivars with a significant difference (p *< *0.05). 

**Figure 3:**
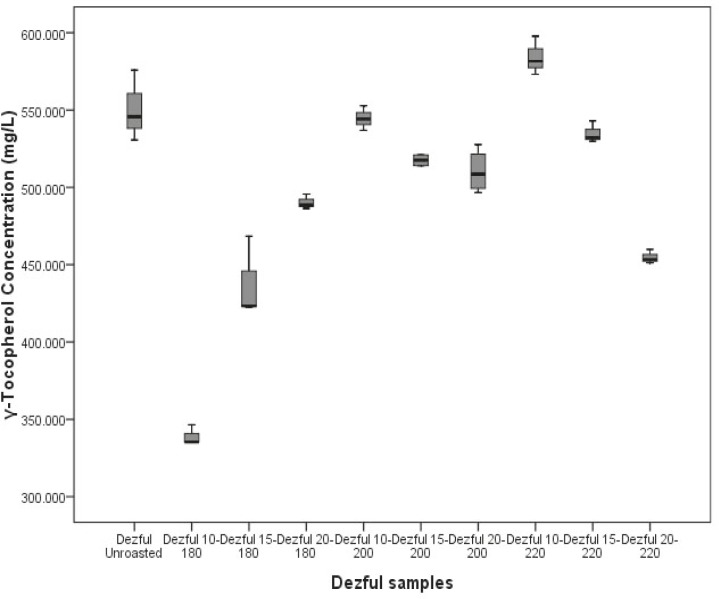
The *γ*-tocopherol content of a sample sesame oil cultivar (Dezful) unroasted and roasted at different time and temperatures

Three cultivars; *Varamin*, *Moghan *and *Karaj *after *Siah *sesame seed oil, have more *γ*-tocopherol content (p *< *0.05) rather than the other four cultivars (Figure 2). A systematic comparisons among the *γ*-tocopherol content of *Dezful *sesame oil cultivar, roasted at different times and temperatures in the range of 10 to 20 min and 180 to 200 °C was also shown in Figure 3. 

*Roasting effects on total phenolic content*

The total phenolic compound in methanolic extract of 8 cultivars of Iranian sesame seeds was measured before and after roasting at 180 °C, 200 °C, 220 °C for 10, 15, 20 min (n=80). TPC increased significantly with the roasting temperature. The amount of TPC varied in different sesame cultivars from 20.109 ± 3.967 μM to 129.300 ± 3.493 μM in *Varamin *and *Naz-Branching *cultivars, respectively, also TPC increased from 70.953 ± 5.863 μM in unroasted Branching Naz as a control to 129.300 ± 3.493 μM after roasting in 200 °C for 20 min (Table 3). *Naz-Non Branching *and *Naz- Branching *cultivars had more total phenolic compound than the others (p *< *0.001), but other cultivars had no significant difference with each other. The results show that the amount of TPC increased significantly as the roasting temperature and time; until 200 °C for 20 min, and they will be decreased by roasting at 220 °C, so the highest activity and content will be achieved by roasting at 200 °C for 20 min. 

**Table 3:** The effect of seed roasting at 200 °C for 20 min on the total phenol content of sesame seeds (mean ±SD, μmol/mL).

**Roasting condition**	**Unroasted**	**200 **°**C ** **20 min**
Cultivar		
Darab	20.31 ± 2.29	98.41 ± 2.14
Varamin	20.10 ± 3.96	102.43 ± 3.05
Non Branching Naz	22.32 ± 1.36	106.52 ± 3.89
Branchj Naz	70.95 ± 5.89	129.30 ± 3.49
Karaj	24.53 ± 3.38	88.97 ± 2.11
Moghan	25.53 ± 3.11	93.86 ± 3.76
Dezful	26.33 ± 2.51w	108.46 ± 3.11
Black sesame	28.95 ± 5.48	110.66 ± 5.41

The high oxidative stability of sesame oil is due to the presence of a large quantity of endogenous antioxidants and phenolic compounds (14). Some studies were undertaken to evaluate the effects of seed roasting conditions on the antioxidant activity. Jeong study agrees with our results, as the total phenolic content, radical scavenging activity, reducing powers, and antioxidant activity of sesame meal extract increased; and several low-molecular weight phenolic compounds were newly formed in the sesame meal after roasting sesame seeds at 200 °C for 60 min (16). 

The relatively greater oxidative stability of oils from roasting treatment which was observed in seeds may be resulting from the formation of some antioxidants (sesamol) from the degradation of other native compounds; sesaminol (17). Sesame oil prepared from roasted sesame seeds has a distinctive flavor and longer shelf-life. Kim believes that the storage stability of unroasted sesame oil is low, but roasting of sesame seed at 170 °C or higher significantly increased the stability of sesame oil. The highest stability was achieved by roasting at 200 °C (18). 

The variation in *γ*-tocopherol contents of sesame seeds may be due to genetic differences or the geographical origin of the seeds (19). The *γ*-tocopherol has a lower vitamin E value in biological systems (20) than the *α*-tocopherol but it is a more potent antioxidant in oils, so γ-tocopherol was examined in our study. *Fukuda et al. (*1*) have suggested that the main active antioxidative constituent in fresh sesame oil extracted from roasted seeds is γ-tocopherol*. According to the results shown in Table 2, not only the contents of *γ*-tocopherol were still retained at more than 80% after roasting for 20 min at all temperatures, as reported in some studies (21-22), but also observed was a significant increase (p *< *0.05) in *γ*-tocopherol contents after heating treatment in the most of the samples. This increase suggests that a high amount of *γ*-tocopherol was bound to membrane proteins or linked to phosphate or phospholipids which heating treatment may break these bonds so that *γ*-tocopherol will release as *Moreau et al. *reported the same for *γ*-tocopherol in corn hulls (23).

As shown in Figure 3, content of *γ*-tocopherol was more in 220 °C for 10 min. The highest *γ*-tocopherol (p *< *0.05) in *Darab*, *Dezful*, *Karaj*, *Moghan*, *Naz-Branching*, *Naz-NonBranching*, *Siah *and *Varamin *samples was seen at 220 °C for 15 min, 220 °C for 10 min, unroasted, 200 °C for 10 min, 200 °C for 10 min, 220 °C for 10 min, 200 °C for 10 min and 200 °C for 10 min, respectively. These data imply that the best time and temperature to achieve the high *γ*-tocopherol in sesame oil is 10 min and 200 °C.

Gertz reports that heating the sesame seeds up to 180 °C could increase the amount of *γ*-tocopherol, however, higher temperatures may cause the inverse effect (24). Additionaly, little has been mentioned on how seed roasting temperature and time affect the sesame oil quality, but the last decade study was done to demonstrate effects of sesame seed roasting temperature and time on its quality characteristics (22). When the roasting time was fixed at about 30 min using a domestic electric oven, the optimum roasting temperature was around 180 °C for the preparation of sesame oil with better quality and flavor (21).

In our study, the most variation after heating with a significant difference (p *< *0.05) between the γ-tocopherol content of the roasted sesame oil cultivars was relevant to *Moghan *and also *Siah *sesame oil samples rather than the other cultivars. There is a relationship between the sesame seeds colors and the content of their *γ*-tocopherol. The black seeds such as *Siah *sesame seeds have the most *γ*-tocopherol content with a significant difference (p *< *0.05) and the white seeds such as *Naz-Branching *sesame seeds have the lowest contents of *γ*-tocopherol.

The results show that the amount of *γ*-tocopherol and TPC increased significantly with the rise in roasting temperature and time until 200 °C, and they are decreased by roasting at 220 °C. The present study showed that Iranian sesame seed can be considered as a good source of natural antioxidants to add to medicines, supplements and foods especially after roasting. The optimum temperature and time of roasting to obtain the most *γ*-tocopherol and total phenolic content is 200 °C for 10 and 20 min, respectively.
